# Plant protection treatments in Switzerland using unmanned aerial vehicles: regulatory framework and lessons learned

**DOI:** 10.1002/ps.8721

**Published:** 2025-02-27

**Authors:** Thomas Anken, Guillaume Coupy, Pierre‐Henri Dubuis, Guillaume Favre, H. Christoph Geiser, Alexandre Gurba, Maria Häni, Markus Hochstrasser, Marco Landis, Tristan Maitre, Christoph Moor, Patrick Mouron, Olivier Sanvido, Yannick Wagner, Jürg A. Zarn, Sebastian L.B. König

**Affiliations:** ^1^ Agroscope, Tänikon 1 Ettenhausen Switzerland; ^2^ Canton of Valais, Department of Economy and Education Sion Switzerland; ^3^ Agroscope, Swiss Federal Agricultural Research Station in Changins Nyon Switzerland; ^4^ Federal Food Safety and Veterinary Office (FSVO) Bern Switzerland; ^5^ Federal Office of the Environment (FOEN) Bern Switzerland; ^6^ Federal Office of Civil Aviation (FOCA) Bern Switzerland; ^7^ Canton of Zurich, Office for Landscape and Nature, Strickhof Lindau Switzerland; ^8^ Federal Office for Agriculture (FOAG) Bern Switzerland; ^9^ State Secretariat for Economic Affairs (SECO) Bern Switzerland; ^10^ Canton of Aargau, Liebegg Agricultural Center Gränichen Switzerland

**Keywords:** Drone, pesticide applications, plant protection products, regulatory framework, unmanned aerial vehicle, unmanned aircraft system

## Abstract

Switzerland was the first European country to approve the use of unmanned aerial vehicles (UAVs) for the purpose of plant protection treatments. The regulatory framework that has been established requires UAV sprayers to undergo a series of technical assessments that have generated a substantial amount of data. Here, we first describe the regulatory framework and the underlying rationales, followed by analyzing data from the technical assessments. The results show that the UAV sprayers being used in Switzerland achieve homogeneous transversal spray liquid distributions with coefficients of variation below 15 % at effective swath widths that are typically lower than those indicated by UAV manufacturers. Moreover, the lateral wind generated by the UAV sprayers as measured at distances of 10 m and 20 m, respectively, is not substantially affected by UAV size or weight. A survey we conducted to gain insight into agricultural practices under the current regulatory framework suggests that up to 11.5 % of the total of Swiss vineyards were treated with UAV sprayers in 2023. Other uses, such as spreading slug pellets, seem to gain importance as well. Finally, efficacy trials performed in Swiss vineyards suggest that UAV sprayers achieve limited control efficacy of powdery and downy mildew at high disease pressure, which is likely to be due to the relatively low amount of deposit around and on the bunches. We conclude this paper by outlining future regulatory challenges and directions for further development. © 2025 The Author(s). *Pest Management Science* published by John Wiley & Sons Ltd on behalf of Society of Chemical Industry.

## Graphical abstract

Unmanned aerial vehicles (UAVs) are increasingly used for plant protection treatments. We describe the corresponding Swiss regulatory framework. We also summarize practical experiences gained from technical assessments performed within this framework, field use patterns and the results of field efficacy trials.

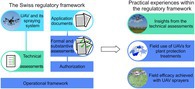

## 1 INTRODUCTION

Switzerland's topography is dominated by mountain ranges belonging to the Alps and their foothills as well as the Jura Mountains, which overall cover approximately 70 % of the country's total area. Due to sloped and rugged terrain, agriculture in mountainous regions is challenging. Traditionally, plant protection products have been applied manually in these difficult‐to‐access areas using hand lance and knapsack sprayers or through applications with manned helicopters. Manual applications are, however, labor intense and lead to significant operator exposure, whereas applications with helicopters are noisy and prone to generating droplet drift, *i.e*., droplets of spray liquid moving to off‐target sites at the time of treatment or soon thereafter.^1^, ^2^, ^3^, ^4^ As a consequence, interest for unmanned aerial vehicles (UAVs) as an alternative to the above‐mentioned application techniques emerged early in Switzerland.

The ISO standard 23117–1 refers to a UAV as an unmanned aircraft system (UAS).^5^ According to the same document, a UAV/UAS corresponds to an ‘aircraft and its associated elements which are operated remotely or automatically’.^5^ For the purpose of spraying, the aircraft needs to be equipped with an unmanned aerial spraying system (UASS), which includes hardware such as spray tank, pump, hoses, nozzles/atomizers as well as hardware and software necessary for the remote control of the application.^5^ A UAV/UAS equipped with a UASS is commonly referred to as a UAV sprayer, which corresponds to the term used in the present work. Please note that the acronym UASS is commonly used as a synonym for a UAV sprayer as well.^6^, ^7^, ^8^, ^9^

In Switzerland, the first application to use a UAV sprayer for plant protection treatments was filed in 2016. Initial treatments with UAVs were performed within the regulatory framework developed for plant protection treatments with manned helicopters.^10^ The latter presently involve Airbus Helicopters H125 B2, which have a maximum take‐off weight of over 2 tons. They typically fly at speeds of approximately 15 m s^−1^ and altitudes of 5–10 m during treatments. To mitigate the risks associated with such heavy machinery, the corresponding regulatory framework stipulates that plant protection products must be specifically authorized for the purpose of aerial spraying. Moreover, it involves stringent safety distances derived from an appropriate risk assessment and a specific authorization is required for each agricultural plot to be sprayed. Considering that (i) the maximum take‐off weight and operating speed of UAV sprayers are lower than those of manned helicopters and (ii) UAV sprayers fly at lower altitudes,^11^ it appeared rather cautious to evaluate the risks of the initial UAV‐based plant protection treatments within a regulatory framework tailored to manned helicopters.

Around 2016, several Swiss governmental institutions expressed the intent to further their understanding of UAV sprayer technology. As a consequence, a 2‐year research project (‘*Vidrone*’) was initiated in which a total of six public agencies were involved. The project goals concerned spray drift, spray coverage, efficacy, airflow and other relevant technical aspects in order to devise a standardized approach to assess UAV‐based plant protection treatments. A further goal that was included at a later stage was related to bystander exposure, defined here as the exposure of individuals whose presence is neither related to UAV flight operations nor to work involving plant protection products.^12^ Based on the results of these trials and subsequent analyses, a regulatory framework tailored to plant protection treatments using UAVs was developed and put into force in 2019 as the first of its kind in Europe. Until today, it has been revised several times taking into account the practical experience at the national level, the results of additional field trials as well as the revision of European unmanned aviation law.^13^, ^14^

The current Swiss regulatory framework relates to (i) how UAVs to be used for plant protection treatments are approved (technical assessments), (ii) how individuals or legal entities may obtain an authorization for UAV‐based plant protection treatments (authorization process) and (iii) how plant protection treatments should be performed (operational framework). The regulatory framework and the specific assessments performed by public agencies in this context are described in the following section.

## 2 REGULATORY FRAMEWORK

### 2.1 Technical assessments

A UAV sprayer to be used for plant protection treatments must undergo a number of technical assessments. Here, the assessment is two‐tiered: First, the UAV sprayer model must be approved. This approval is referred to as ‘homologation’ and only needs to be performed once per UAV sprayer model. To determine whether a UAV model and its associated spraying system should be homologated, Agroscope, which is the competent homologation authority, works from a number of requirements. These relate to nozzles/atomizers, transversal spray distribution, flight precision, pump(s), tubing, tank, agitation system, lateral wind generated by the UAV, external pressure gauges and built‐in strainers.^15^ Here, an on‐board agitation system is not mandatory (in fact, UAV sprayers are usually not equipped with such a system).^8^ An external agitation system that can be used when the UAV sprayer is not airborne is deemed sufficient to ensure that a plant protection product contained in the spray tank does not settle out. Regarding the external pressure gauge, it should be possible to connect such a device directly to the liquid circuit of the spraying system without changing the flow rate of the latter.

**Figure 1:**
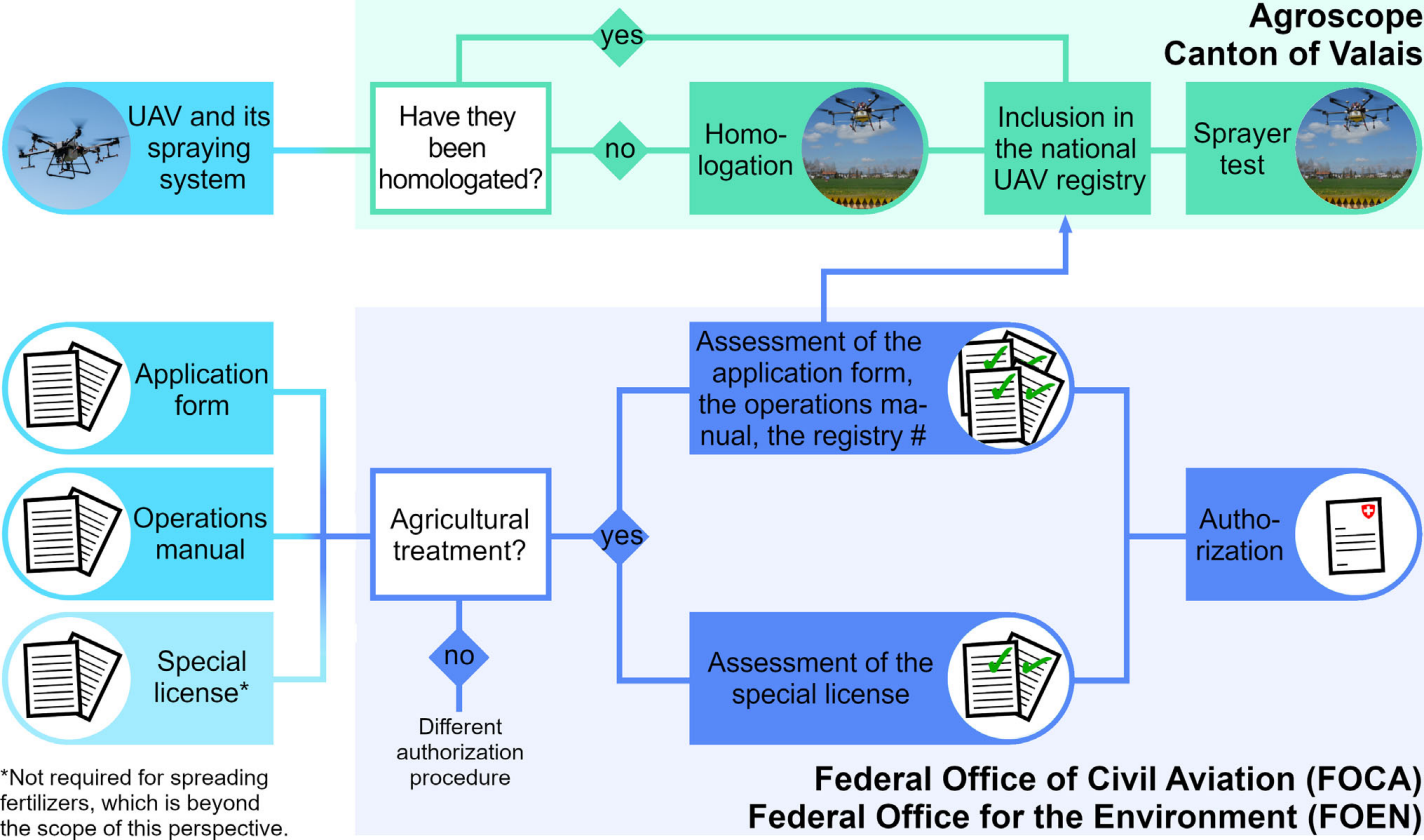
Flow chart outlining the technical assessments and the authorization process associated with UAV‐based plant protection treatments.

Each individual UAV sprayer is included in the national UAV registry upon notification, provided that it corresponds to a model that has been homologated. Based on article 61(5) of the Swiss Plant Protection Ordinance,^16^ each individual UAV sprayer must, however, pass a sprayer test before it can be put into service and every 3 years thereafter. The sprayer test constitutes the second step of the technical assessment and relates in principle to the same requirements as the tests performed for the purpose of homologation with two exceptions: (i) lateral wind speed measurements are not performed and (ii) the precision of the flying route is not measured. It is instead visually estimated whether the UAV respects a precision of ±50 cm while flying over georeferenced poles.^15^ So far, these sprayer tests have been conducted by Agroscope or the Canton of Valais. Please refer to Fig. 1 (top) for a graphical representation of the relevant processes. Maintenance work performed by the UAV owner is beyond the scope of these tests.

### 2.2 Authorization process

UAV‐based plant protection treatments require an authorization issued by the Federal Office of Civil Aviation (FOCA). The authorization process is summarized in Fig. 1 (bottom). An authorization application involves a risk assessment, which since 2023 formally corresponds either to (i) a ‘Pre‐Defined Risk Assessment’ or (ii) a ‘Specific Operations Risk Assessment’, depending on whether or not FOCA has already performed the risk assessment for the given use scenario, followed by publishing it as an acceptable means of compliance to Article 11 of Regulation (EU) 2019/947.^13^, ^17^ In both cases, an application form must be submitted along with an operations manual and a special license. The latter is a prerequisite for handling plant protection products in an occupational setting as per Article 7 of the Swiss Chemical Risk Reduction Ordinance.^18^ The validity of the specialist license is assessed by the Federal Office for the Environment (FOEN). The application form and the operations manual are assessed by FOCA, which also checks whether the UAV sprayer has been tested as described in the previous subsection. Authorizations are valid for 1–2 years.

### 2.3 Operational framework

**Figure 2:**
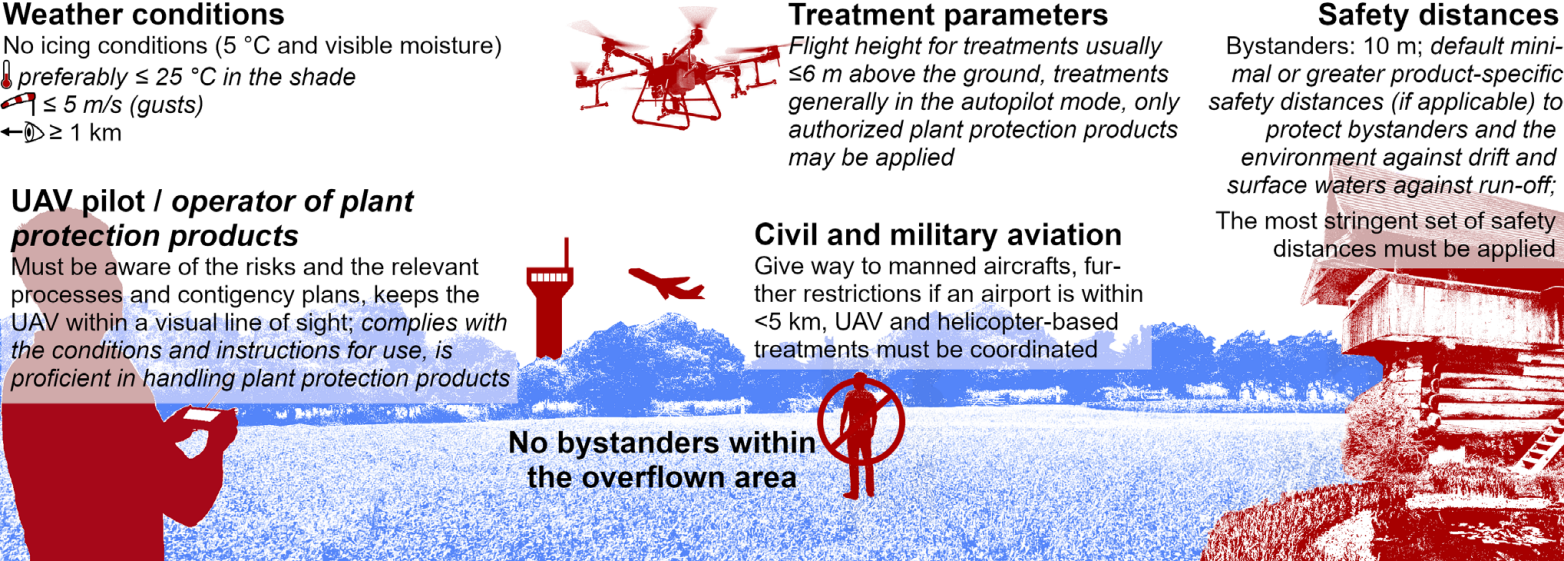
Key aspects of the operational framework for UAV‐based plant protection treatments. Aviation and aircraft‐specific requirements are indicated in Roman font, plant protection‐specific aspects are italicized.

This section summarizes key points of the operational framework, which are also illustrated in Fig. 2. Please note that the framework draws from two legal areas, *i.e*., aviation and plant protection, which is also highlighted in Fig. 2. The entire framework is described elsewhere by the Federal Office of Civil Aviation.^19^

#### 2.3.1 General

Plant protection products must be authorized before they can be put on the market. The criteria for authorization are laid out in the Swiss Plant Protection Ordinance and largely correspond to those described in the European Regulation (EC) No 1107/2009.^16^, ^20^ In this context, a number of risk assessments are performed that typically relate to applications with boom and/or air‐assisted ground sprayers. The results of the *Vidrone* project (see Section 1) and follow‐up field studies addressing environmental and bystander exposure suggest that drift mitigation measures for UAV‐based treatments can be derived from assessments done for the above‐mentioned ground sprayers.^6^ As a consequence, all plant protection products authorized for treatments with conventional ground‐based equipment may also be applied by UAV. Analogous to ground‐based treatments, UAV‐based treatments do not require a specific authorization per plot to be treated. This regulatory approach differs fundamentally from that related to treatments with manned helicopters (see Section 1).

#### 2.3.2 UAV remote pilots and authorization holders

Pilots must be aware of the risks associated with UAVs and assess these with standard processes to be defined beforehand. These processes have to consider, for example, the preparation for a flight, the zone to be overflown and the corresponding safety buffers, and aim to ensure that operations are performed in a controlled fashion. Authorization holders, which may be pilots but can also be legal entities, should have procedures in place for handling unforeseen situations. Specifically, the operations manual must include an emergency response plan that addresses the safety risks associated with loss of UAV control and describe how the impact of possible accidents can be mitigated. Authorization holders must document all flights.

#### 2.3.3 Operators

Operators, defined here as persons who are involved in the application of plant protection products,^12^ must comply with product‐specific conditions and instructions for use when preparing the spray liquid and cleaning the tank. They must further hold a specialist license or must have been instructed accordingly by a license holder (see Authorization process section).

#### 2.3.4 Flight parameters

Flight heights during treatments should not exceed 6 m above the ground, though, greater flight heights are permitted when high fruit trees are to be treated. In general, flight routes must be planned in advance and flown in the autopilot mode. Human intervention is permitted for take‐off, landing, spot treatments with herbicides and additional spot treatments following broadcast applications. It must be possible to interrupt the spraying process at any time and control the aircraft manually.

#### 2.3.5 Protection of bystanders and the environment

Bystanders are not allowed in the zone to be overflown. For UAVs with a maximum take‐off weight greater than 10 kg, which applies to all UAV models homologated to date (see Insights from the technical assessments section), a safety distance of at least 10 m must be kept between the UAV and bystanders in the surrounding area. Further risk mitigation measures relate to pesticide drift. To mitigate the risk of drift, a minimal safety distance of 20 m must be maintained from surface waters, biotopes, the property boundary of buildings, public places, residential areas and bystanders during agricultural operations when field crops, grassland and meadows, vegetables, strawberries or lawns are treated or when herbicide treatments are performed. There is no default minimal safety distance to mitigate the risks associated with drift with respect to other use scenarios. Safety distances for drift mitigation can, however, also be specified in the conditions for use of the product and apply instead of default safety distances if they are greater. Finally, plant protection products may cause surface water contaminations through run‐off. A minimal safety distance to surface waters of 3 m has to be kept according to the Swiss Chemical Risk Reduction Ordinance.^18^ A minimal distance of 6 m has to be kept by farmers that are supported according to the Swiss Direct Payments Ordinance.^21^ Moreover, product‐specific risk mitigation measures are defined in the corresponding authorizations.^22^ Overall, the most stringent set of safety distances has to be applied.

#### 2.3.6 Civil and/or military aviation

In a number of cantons, plant protection treatments may be performed by manned helicopters. Here, authorization holders must coordinate their operations with the service provider for helicopter‐based plant protection treatments pre‐flight. In case of aircraft proximity, manned aircrafts have priority over UAV sprayers and the latter must immediately cancel their flight. Special rules apply when the runways of a civil or military airport are within a distance of less than 5 km of the plot to be treated.

#### 2.3.7 Weather conditions

It is recommended that air temperature in the shade should be less than 25 °C during operations and flights under icing conditions are prohibited. Moreover, spraying in the presence of wind gusts exceeding 5 m s^−1^ are prohibited and spraying should be avoided at wind speeds greater than 3 m s^−1^. Finally, visibility should be greater than 1 km to permit direct eye contact with the UAV at all times.

## 3 INSIGHTS FROM THE TECHNICAL ASSESSMENTS

**Figure 3:**
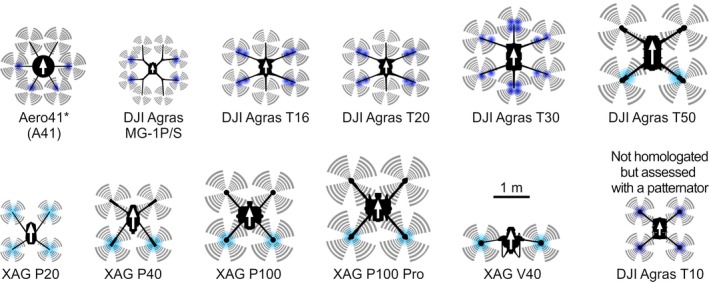
UAV models and their associated spraying systems that have been homologated in Switzerland until August 2024. The front of each UAV is indicated with a white arrow. Nozzle/atomizer positions are indicated by colored circles (dark blue = hydraulic nozzle, light blue = rotary atomizer). The scale applies to all UAV models shown. *Technically similar models are referred to as HSE AG‐V6A and AgroFly SpUAV and are also used in Switzerland. Please note that the HSE AG‐V6A can in principle be equipped with a boom to which spray nozzles are attached,^23^ but it was not homologated in this configuration.

Until August 2024, 11 UAV models and their associated spraying systems have been homologated and 90 individual UAV sprayers have been included in the Swiss national UAV registry. As shown in Fig. 3, all homologated UAV sprayer models are characterized by nozzles/atomizers located directly underneath or in close proximity to the rotors. As described in Technical assessments section, the Swiss authorities perform a number of technical assessments in the context of the regulatory framework. Results of these assessments were subjected to a meta‐analysis, the findings of which are summarized in the following subsections. These analyses have not been published elsewhere.

### 3.1 Transversal distribution of the spray liquid

Spray patternators permit to analyze the transversal spray liquid volume distribution that an agricultural boom sprayer generates in a single pass.^24^ They are also used in the context of the technical assessments of UAV sprayers performed in Switzerland. In general, spray patternators consist of horizontally mounted grooves that channel the spray liquid into collecting cylinders. During the tests done as part of the regulatory framework of UAV‐based plant protection treatments, the UAV sprayer maintains a fixed hovering position typically 2.5 m above the center of the patternator.^25^ The nozzles mounted onto the UAV sprayer to be tested or the settings of the rotary atomizers, respectively, are proposed by the owner of the machine to reflect typical agricultural practice. Such stationary spray patternator measurements have recently been demonstrated to yield similar results as dynamic single‐pass spray pattern analyses using water‐sensitive paper or tracer collected on filter paper.^26^ In practice, UAV‐based plant protection treatments rarely involve, however, a single pass. Instead, they typically involve multiple back‐and‐forth passes where treatment lanes are spaced by a certain distance (‘swath width’). To simulate transversal spray liquid volume distributions resulting from a back‐and‐forth pattern at varying swath widths, distributions measured in patternator experiments are duplicated and increasingly offset from each other.

The generally accepted measure of uniformity of a spray liquid distribution is the coefficient of variation (CV). It is calculated as the standard deviation divided by the arithmetic mean multiplied by 100 %.^27^ In the context of the technical assessments, the CV is calculated for all simulated liquid volume distributions. In the ISO standard 16 122–2, which relates to the inspection of horizontal boom sprayers, a CV below 10 % is stipulated.^27^ However, UAV sprayers are known to produce sprays that are more variable than those produced by ground sprayers.^9^ Therefore, the effective swath width, also referred to as the effective working width, that is determined in the context of the Swiss regulatory framework for UAV‐based plant protection treatments is defined as the greatest inter‐lane spacing that produces a deposition profile with a CV lower than 15 %. Technical details on how effective swath widths are determined have been published elsewhere.^26^

**Figure 4:**
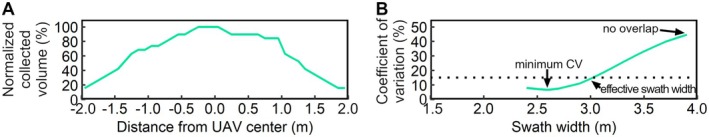
Results of a patternator experiment and data analysis. A Typical liquid volume distribution of a DJI Agras T20. The data represented are expressed as a percentage of the highest measured volume. B Spray pattern uniformity at varying swath widths with respect to simulated distributions built from the data shown in panel (A). The black dotted line corresponds to a coefficient of variation of 15 %.

Figure 4 depicts the typical results of a patternator measurement as well as the effect of swath width on the CV of simulated liquid volume distributions computed from the experimental data. In this example, the tank was fully loaded with water at the beginning of the experiment, which corresponds to established practice in the context of the Swiss homologation procedure. A total volume of 4.88 L was collected. This means that the difference between the UAV weight at the beginning and at the end of the experiment was approximately 5 kg. As shown in panel B, the highest CV corresponds to roughly 40% and is observed when there is no overlap between adjacent distributions, which would correspond to a flight line spacing of 3.8 m in a multiple pass spray pattern. The simulated distributions are characterized by a CV below 15% when flight lines are spaced by 2.4–3.0 m, with a minimum of approximately 8% at 2.6 m and an effective swath width of 3.0 m. Defining the effective swath width as the largest swath width with a CV below 15% is regarded as a compromise between treatment efficacy and efficiency. It should be noted that higher CVs have been discussed outside the context of the Swiss regulatory framework.^28^ Less uniform spray patterns are typically associated with larger swath widths, and hence, an increase of the surface that can potentially be treated in a given time period. However, higher CVs also increase the risk of encountering undesired effects, such as decreased efficacy of the treatment against the target pest(s) and potential phytotoxicity against the crop one intends to protect.

### 3.2 Effective swath widths

**Figure 5:**
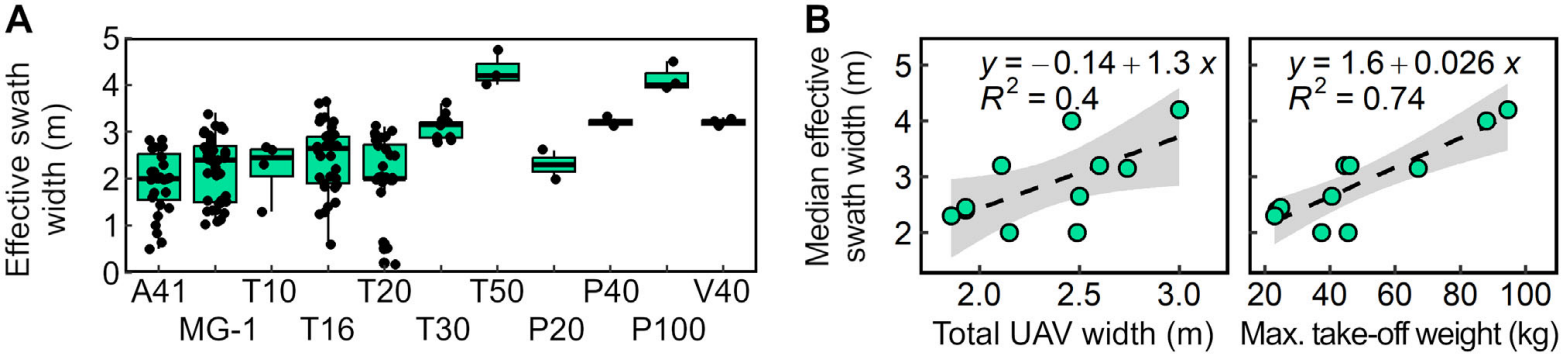
Effective swath widths of UAV sprayers homologated in Switzerland. (A) Box‐and‐whisker plot summarizing the results of the technical assessments per UAV model. The upper and lower hinges of a box correspond to the 75^th^ and 25^th^ percentile of the dataset, respectively. The median is indicated as a bold line. Whiskers extend from the respective hinge to the largest and smallest value of the dataset, but no further than 1.5 times the distance between the 25^th^ and 75^th^ percentile, respectively. Individual values are indicated as black jitter. (B) Relationship between measured total UAV width including propellers (left) or measured UAV maximum take‐off weight (right) and median effective swath width. Each dot corresponds to a UAV‐specific value, dashed lines correspond to the linear regression equations indicated. Standard errors are represented as grey swaths.

Figure 5(A) shows the distribution of 158 effective swath widths, all of which were determined as described in Transversal distribution of the spray liquid section. In general, the experimentally determined values are lower than those specified by the manufacturers. For example, a median effective swath width of 2.7 m was determined for the Agras T16, whereas the manufacturer indicates a spray width of 4.0–6.5 m at a flight height of 1.5–3.0 m above the crop.^29^ Here, it is important to point out that high deposition variability can have undesired effects (see Transversal distribution of the spray liquid section).

All UAV sprayers that have been tested in the context of the Swiss regulatory framework are characterized by nozzles/atomizers located directly underneath or in close proximity to the rotors (Fig. 3). For these configurations, the effective swath width is expected to be largely governed by the width of the UAV considering the downwash generated by its propellers at a given flight height.^26^ Interestingly, UAV maximum take‐off weights correlate more strongly with median effective swath widths than do total UAV widths (Fig. 5(B)). This could be due to the increased downwash that is required to generate a sufficient lift force to support the weight of heavy UAVs. However, even the heaviest UAV sprayer assessed does not exceed an effective swath width of 5 m. Rather narrow swath widths of UAV sprayers have been found in independent studies performed outside Switzerland. For example, swath widths of 2.0–2.5 m, 3 m and a little less than 5 m have been reported for a HexAero prototype hexacopter, the XAG P20 and the PrecisionVision 35, respectively.^30^, ^31^, ^32^ It should be emphasized, however, that very different swath widths would likely be observed for UAV sprayers equipped with horizontal booms.

The patternator data discussed in the present work were collected in experiments during which UAV payload was maximal at the beginning, followed by a gradual decrease. As the weight of the UAV sprayer decreases, the downwash decreases.^33^ Specifically, it was shown in simulations involving a hovering multicopter that payload primarily impacts downwash velocity, albeit the shape of the downdraft field was less affected.^34^ Consequently, the data generated in the patternator measurements performed within the Swiss regulatory framework for UAV‐mediated plant protection treatments were recorded over a range of downwash airflows. As such, they do not allow to assess whether, and if so, to what extent payload‐dependent fluctuations in downdraft airflow affect the swath width of UAV sprayers. We agree with other researchers who have called for further research on this topic.^33^

The patternators used to generate the data analyzed herein were horizontally mounted. Similarly, dynamic spray pattern measurements with UAV sprayers are typically performed on flat surfaces.^26^, ^31^, ^32^ Hence, these experiments do not account for potential interaction between the application equipment and the crop canopy, which might occur during plant protection treatments in vegetated areas. Direct transposition of patternator data to plant protection treatments in vegetated fields is generally accepted for boom sprayers. With regard to UAV sprayers, the Organisation for Economic Co‐operation and Development has recently pointed out, however, that ‘it is not known if the force of downwash affects the amount of pesticide intercepted by a crop and reaching the ground, or if subsequent passes dislodge any previously deposited spray from leaves’.^28^ We therefore encourage further research on downwash‐crop interaction and the transposability of swath width measurements to plant protection treatments in vegetated areas.

### 3.3 Lateral wind speeds

All UAVs that have been homologated in Switzerland until August 2024 carry several rotors (Fig. 3). Rotors are required for lift, but may also generate turbulences and wingtip vortices, resulting in lateral wind components.^33^ Lateral winds transport droplets away from the downwash field, thereby reducing the UAV's ability to draw them to the ground and increasing the risk of drift.^33^ To quantify to what extent UAV airflow may contribute to drift, lateral wind measurements were performed in an open field. During these tests, UAVs followed a single line at a speed of 3 m/s and a height of 2.5 m above the ground. Concomitantly, wind speeds were monitored at a frequency of 10 Hz for a duration of 50 s using tridimensional anemometers arranged parallel to the flight line located at a lateral distance of 10 m and 20 m and a height of 1 m and 2 m, respectively. Further methodological details are provided elsewhere.^25^ In this experimental setup, ambient wind constitutes a potential confounder the results were not corrected for. However, care was taken to conduct all experiments at low ambient wind speeds by consulting wind forecasts beforehand.

**Figure 6:**
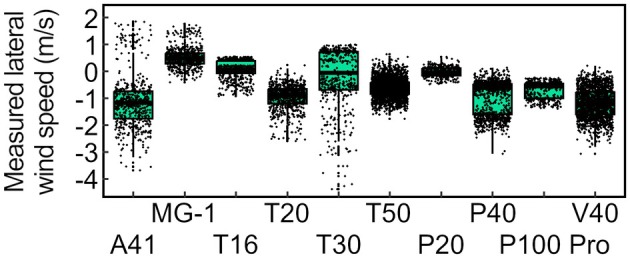
Box‐and‐whisker plot summarizing the results of lateral wind speed measurements performed with different UAV sprayers. All data shown were collected at a height of 1 m above the ground and a lateral distance of 10 m from the UAV. The upper and lower hinges of a box correspond to the 75^th^ and 25^th^ percentile of the dataset, respectively. The median is indicated as a bold line. Whiskers extend from the respective hinge to the largest and smallest value of the dataset, but no further than 1.5 times the distance between the 25^th^ and 75^th^ percentile, respectively. Individual values are indicated as black jitter.

Figure 6 represents the distribution of lateral wind speeds for different UAV sprayers that were measured at a height of 1 m and a distance of 10 m, which corresponds to the sampling point with the highest overall wind speeds. Both positive and negative values correspond to wind blowing perpendicular to the flight line (90° or 270°). The vast majority of wind speeds fall into a range of 0 to −2 m s^−1^. In‐depth analysis of the data did not unveil a correlation with any technical parameter of the UAVs (data not shown). In particular, no association between UAV weight and lateral wind speed was observed. These results suggest that UAV sprayers by themselves may be less likely to cause droplet drift in comparison to ground‐based orchard sprayers, which are typically equipped with fans generating strong upward‐ and/or sideward‐directed airflows.^35^ Indeed, the DJI Agras T30 compared favorably to numerous reference datasets collected for orchard sprayers in a series of field trials.^6^

**Figure 7:**
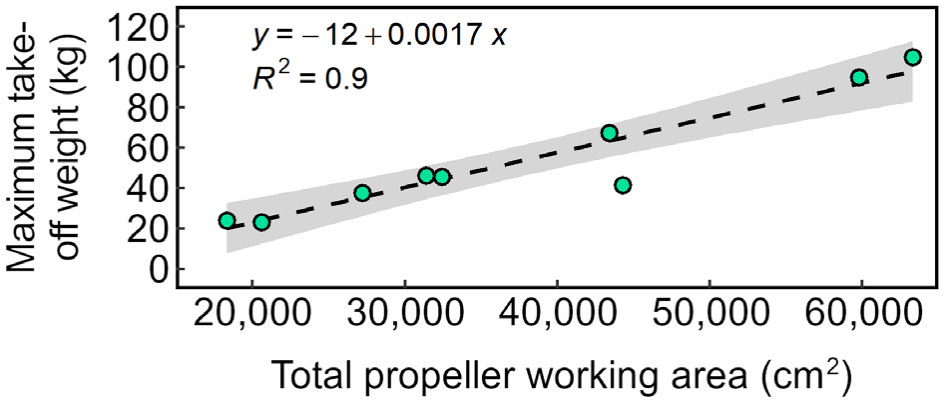
Relationship between total propeller working area and measured UAV maximum take‐off weight of nine different UAVs. Each dot corresponds to a UAV‐specific value, the dashed line corresponds to the linear regression equation indicated. Standard errors are represented as grey swaths.

Finally, considering the A41, MG‐1S, T16, T20, T30, T50, P20, P40 and P100 Pro, we computed the total propeller working area per UAV from the respective propeller diameters. The results were plotted against the maximum take‐off weight (Fig. 7). Interestingly, we observed a strong correlation, even though the UAVs substantially differ with respect to their design and size. The lift force generated by rotary‐wing aircrafts depends on the wing area, the rotational speed of the rotor(s) and the lift coefficient for a given angle of attack.^36^ Hence, our results suggest that rotor speeds may not substantially differ with respect to the UAV sprayers considered under the assumption that there are no tangible differences regarding the lift coefficients. As a caveat, it should be emphasized, however, that the latter were not determined as part of the technical assessments.

## 4 USE OF UAVs FOR PLANT PROTECTION TREATMENTS

UAV‐based plant protection treatments in Switzerland were initially performed within a regulatory framework tailored to manned helicopters that was replaced by a UAV‐specific regulatory framework. There are, however, knowledge gaps as to how UAV use has evolved following this transition. To address this gap, we conducted a survey among all authorization holders as of the beginning of the season of 2024. A total of 11 individuals, *i.e*., 28 % of Swiss authorization holders at the time the survey was conducted, responded to our request. The information provided concerned the area treated using UAVs per year, by crop, by target pest, by canton and by slope. These data have not been published elsewhere. Nonresponse bias analysis revealed that service providers are overrepresented in the survey population. From our experience, service providers tend to treat more hectares per year than individual farmers. We have, however, no indications that treatments performed by service providers and those done by farmers display systematic differences with regard to the other parameters‐of‐interest, *i.e*., the treated crops, the target pests, the geographical distribution of the treated plots and their slopes. Some respondents submitted information on treatments not related to agricultural pest control and/or data related to experimental trials. As these data were outside the scope of the survey, they were excluded. Please refer to the Supporting Information for more details on the methodology of the survey, deviations from the original survey plan, data gaps and how we dealt with the latter to prevent survey attrition.

**Figure 8:**
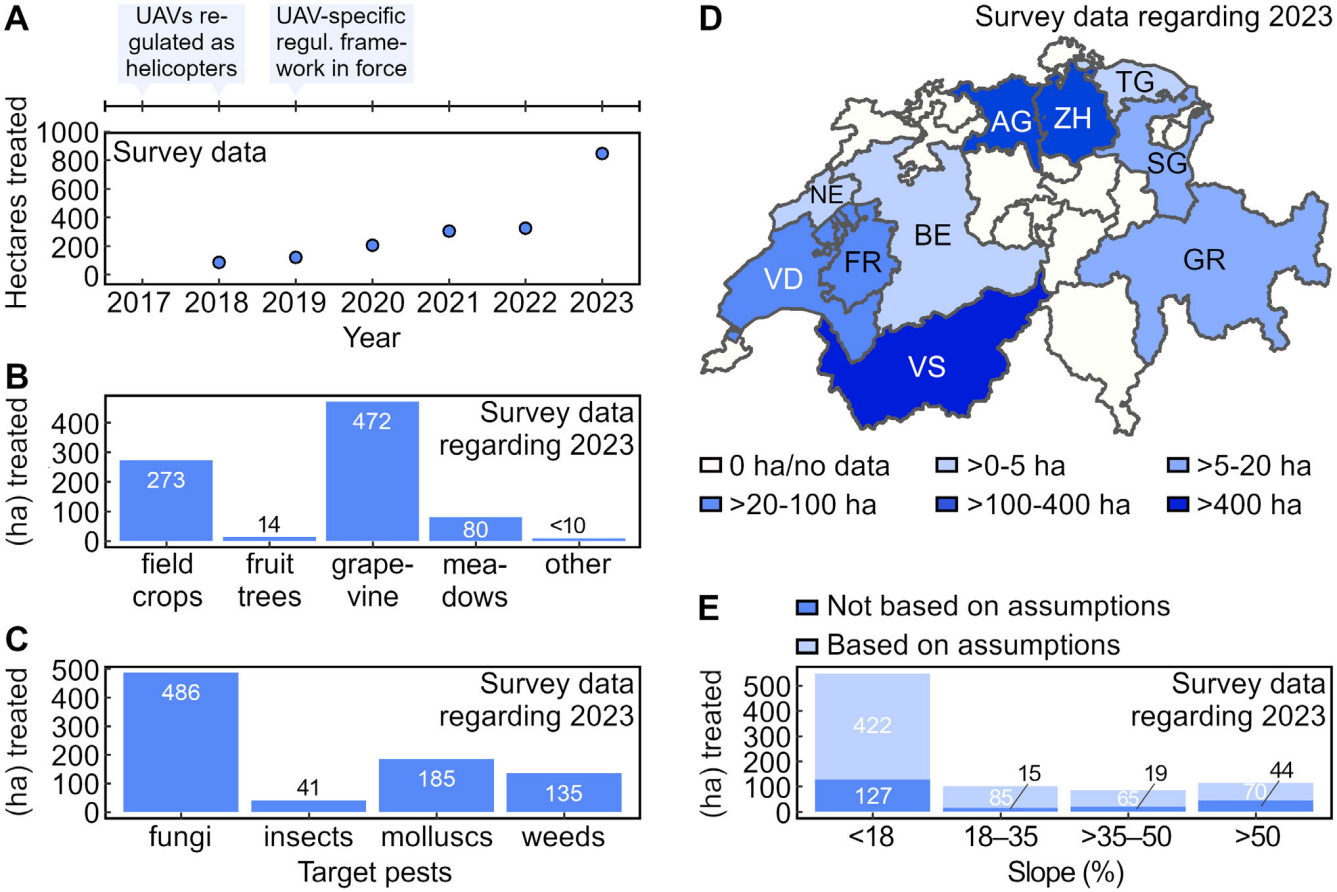
Use of UAVs for plant protection treatments in Switzerland. (A) Total agricultural area treated with UAVs per year. (B, C, E) Agricultural area treated with UAVs in 2023 per crop type, target pest and slope. Values indicated were rounded to integers for clarity. (D) Map of Switzerland showing the agricultural area treated with UAVs in 2023 per canton. Panel based on the open access swissBOUNDARIES3D map.^44^ All panels were built using aggregated data from all respondents. Multiple UAV‐based treatments of the same plot during the same year were considered only once.

Figure 8 depicts the aggregated results of the 11 datasets submitted. None of the values represented in the figure was subject to extrapolation. Figure 8(A) shows the total agricultural area treated with UAVs per year. No data were submitted with respect to the season of 2017. In 2018, a total of 86 ha were treated. In the following years, the total treated area steadily increased and reached a peak of 846 ha in 2023.

In‐depth analyses were performed based on the dataset relating to the season of 2023. As depicted in Fig. 8(B), the respondents indicated that vineyards corresponding to a total area of 472 ha were treated by UAV that year. Most of these treatments involved fungicides (Fig. 8(C)). A second widespread pattern was the treatment of field crops, such as sugar beets, sunflowers, potatoes or canola, with slug pellets (185 ha). Other uses reported for the season of 2023 concerned herbicides for weed control in meadows (80 ha), field crops (52 ha) and grapevine (<1 ha) as well as fungicide treatments of fruit trees (14 ha). Insecticide treatments of various crops were also sporadically performed (<5 ha). Finally, UAVs were also used to release *Trichogramma evanescens* over corn fields (36 ha).

Figure 8(D) shows that the respondents performed UAV‐based treatments in numerous cantons of the Swiss Confederation in 2023. With a treated area of 444 ha, 350 ha of which correspond to vineyards, UAV‐based treatments were widespread in the Canton of Valais (VS). UAVs also appear to be an established means for plant protection treatments in the Cantons of Aargau (AG) and Zurich (ZH). Here, UAVs were mostly used to treat field crops and grapevine. The results of the survey suggest that plant protection treatments with UAVs were performed in at least six other cantons that year.

Figure 8(E) depicts the agricultural area treated by the respondents in 2023 per slope. In multiple instances, neither directly usable data on slopes nor geographical coordinates that permitted to extract slopes were provided. Where usable data were available, they suggested that UAVs were predominantly used to treat plots with slopes <18%. Steeper plots, many of which displaying slopes >50%, were also regularly treated by UAV. This overall picture does not change upon analyzing the remaining available data based on the assumptions described in the Supporting Information.

UAV sprayers constitute an established application technique in East Asia, where they are used, for example, for pest control in rice, wheat, corn and cotton as well as fruit trees.^33^, ^37^ Regarding Europe, Sarri *et al*. previously hypothesized that UAV sprayers could be particularly useful for plant protection treatments in difficult‐to‐access vineyards such as those of the Ribeira Sacra (Spain), Banyuls (France) and the Moselle region (Germany).^38^ However, only little published data are available regarding the actual use patterns of UAV sprayers in Europe. Here, it is of note that within the European Union, of which Switzerland is not a member, aerial spraying is generally prohibited, though, it may be allowed in special cases.^39^ With a total treated area of 471 ha in 2023, UAV‐based plant protection treatments in vineyards of various slopes indeed constituted the most important use pattern among the survey respondents. Particularly in the Canton of Valais, vineyards are typically localized in fragmented agricultural land where individual plots cover only a fraction of a hectare in many cases. Most treatments performed in vineyards targeted fungi. As the survey respondents represent 28 % of all Swiss authorization holders, the treated area can be multiplied by 3.57 (1/0.28) to obtain a national estimate of 1681 ha. In 2023, the Swiss vineyard surface area corresponded to 14 569 ha.^40^ Thus, our results suggest that 11.5 % of the Swiss vineyard surface area may have been treated by UAVs in 2023 although our estimate should be interpreted as an upper bound due to the nonresponse bias described above. Considering that (i) the area treated by helicopter decreased from 1545 ha in 2017 to roughly 900 ha in 2021,^41^, ^42^ and (ii) the plant protection products specifically registered for aerial spraying with manned helicopters exclusively correspond to fungicides for treatments of grapevine and apricot,^43^ our findings further suggest that UAV sprayers may have replaced helicopters for plant protection treatments to a certain extent. Slug control in field crops as well as weed control in field crops and meadows constituted further important use patterns among survey respondents. In our experience, field crops not only tend to be cultivated on less fragmented plots than grapevine in Switzerland, but also on flatter terrain. Hence, our results suggest that Switzerland's rugged landscape and the presence of small sized agricultural plots may have shaped certain use patterns documented in the survey while others have no obvious link to local topology and/or land use.

## 5 RESULTS OF EFFICACY TRIALS

As described in Technical assessments section, the Swiss regulatory framework subjects UAVs and their associated spraying systems to a number of technical assessments. For the Swiss authorities, it was important to determine whether UAVs that passed these assessments achieve acceptable control efficacies under field conditions and whether recommendations could be made with respect to agricultural practice. As a consequence, a series of field trials relating to fungicide treatments in vineyards were performed over the seasons of 2018–2020.^45^ Here, the aim was to quantify the control efficacy of UAV sprayers in the context of a likely area of application and to compare it to that achieved with conventional ground‐based sprayers.

A total of 12 trials were performed on four different experimental vineyards located in the Cantons of Vaud and Valais. Each trial site was subdivided into three sub‐plots that were not treated (‘control’), treated by UAV sprayer (‘UAV’) or treated with a conventional air‐blast or knapsack sprayer (‘ground’), respectively. Four trials involved a fourth sub‐plot on which plant protection treatments were done with a UAV sprayer but one to two additional ground‐based applications were also performed at BBCH 73–75 (‘UAV + ground’). The frequency and severity of downy and powdery mildew infestations before harvest were assessed by visual inspection of leaves and bunches. Further details on the methodology and unforeseen events during the trials are described in elsewhere^45^ and in the Supporting Information.

**Figure 9:**
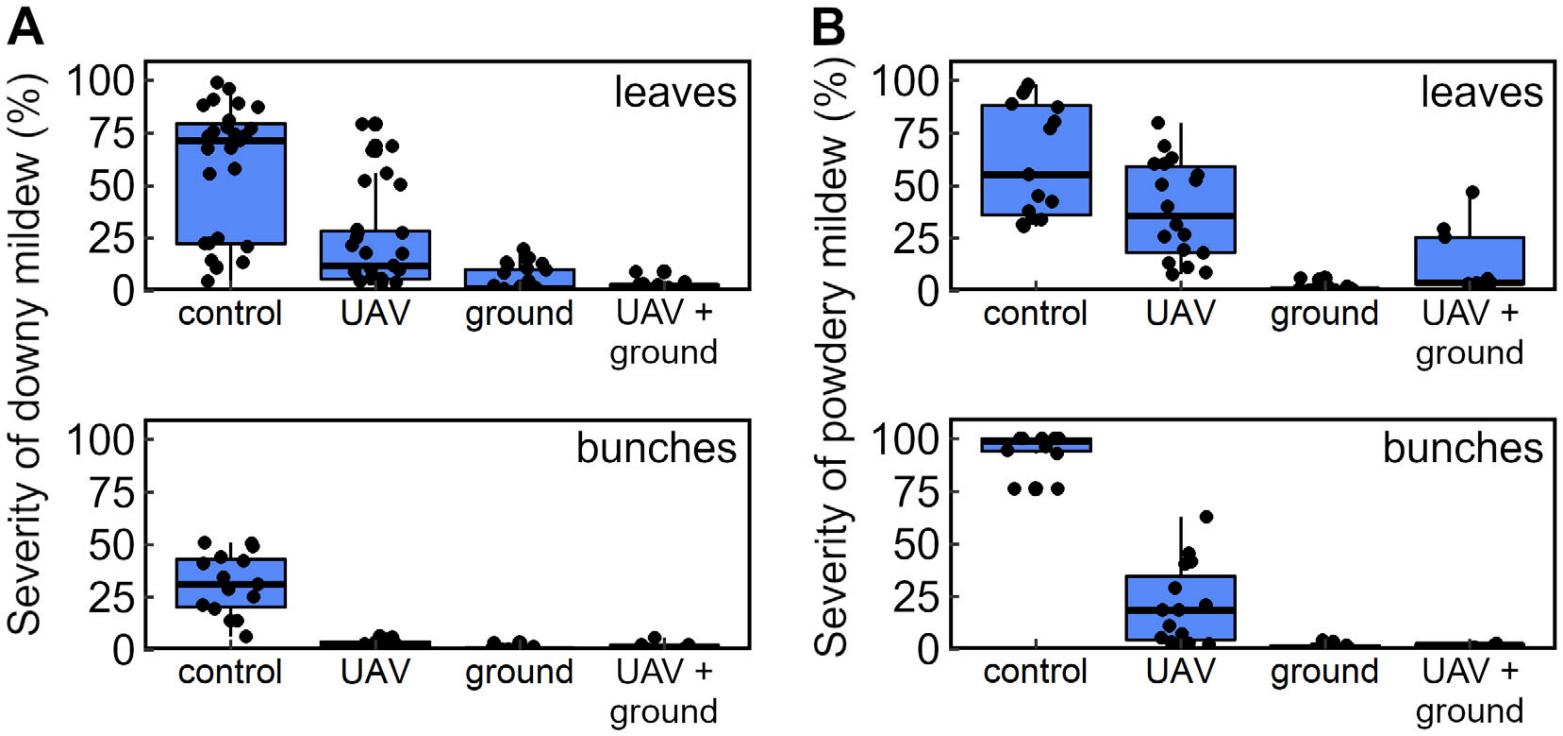
Box‐and‐whisker plots of mildew occurrence in trials conducted in Swiss vineyards from 2018 to 2020. (A) Severity of downy mildew on leaves (top) and bunches (bottom). (B) Severity of powdery mildew on leaves (top) and bunches (bottom). In each box plot, the upper and lower hinges of a box correspond to the 75^th^ and 25^th^ percentile of the dataset, respectively. The median is indicated as a bold line. Whiskers extend from the respective hinge to the largest and smallest value of the dataset, but no further than 1.5 times the distance between the 25^th^ and 75^th^ percentile, respectively. Individual values are indicated as black jitter. All plots are based on published data.^45^

The results are shown in Fig. 9. Negative controls showed that the disease pressure from powdery mildew was overall higher than from downy mildew. Independent from pathogen‐specific differences in disease pressure, UAV‐based fungicide treatments of grapevine were generally observed to be less efficacious than those performed with conventional spraying equipment. A fluorescent tracer was added to the tank mix in one of the efficacy trials. The tracer permitted to visualize the coverage using a UV light source and to quantify spray deposition in different regions of the leaf canopy and on bunches. Deposition was quantified fluorometrically following tracer extraction with an organic solvent.^6^ Analytical results were expressed as ng tracer/cm^2^ foliage or bunch. As the volume rates were different, *i.e*., 123 L ha^−1^ (UAV) as opposed to 378 L ha^−1^ (backpack sprayer), the results were further normalized with respect to the respective application rate of the tracer to allow for a comparison. While the normalized amount of deposit in the upper part of the canopy was comparable for both application techniques tested, UAV‐based treatments resulted on average in 3.6 times less deposit on leaves in proximity of the bunches and 7.1 times less deposit on bunches.

Overall, the results suggest that UAV‐based treatments need to be performed in conjunction with ground applications targeted to the bunches after fruit set to obtain satisfactory mildew control levels in grapevine, in particular when disease pressure is high.^45^ Inferior control efficacy achieved by UAV sprayers alone has been described in independent reports relating to wheat mildew control.^46^ With regard to the results of the trials performed in Switzerland, the limited control efficacy is likely to be due to the relatively low amount of spray deposit around and on the bunches. Preferential spray deposition in the upper regions of the canopy following UAV‐based plant protection treatments has been observed by others in independent trials performed in vineyards and orchards.^47^, ^48^ In a recently published review, the lower efficacy of UAV sprayers compared to conventional techniques operating at higher volumetric application rates against fungal pathogens is reiterated and the need for good coverage, especially with contact fungicides, is emphasized in this context.^49^ Interestingly, it is stated in the same paper that control achieved with UAV sprayers has been shown to be comparable to that of conventional equipment when the target pest was an insect, as pest mobility reduces the need for good coverage.^49^ Based on the results of the survey, insect control does, however, not appear to be a common use scenario for UAV sprayers in Switzerland at present. Comparatively low deposits and leaf coverage resulting in severe powdery mildew infestation at high disease pressure have also been reported with respect to fungicide treatments performed with manned helicopters.^50^ This indicates UAV sprayers and manned helicopters may share similar limitations with regard to treatment efficacy.

## 6 CONCLUSIONS AND OUTLOOK

Swiss authorities have applied a regulatory framework for UAV‐based plant protection treatments over the past years. This framework subjects UAVs and their associated spraying systems to a number of technical assessments in the context of which a considerable body of data has been generated. Based on this dataset, we performed a meta‐analysis. The results suggest that UAV manufacturers may overestimate effective swath widths when multiple back‐and‐forth passes are considered and a coefficient of variation below 15 % is used as a requirement. Multiple back‐and‐forth passes are widespread agricultural practice when crops are treated with UAVs in our experience, and we regard the threshold of 15 % as a pragmatic compromise between treatment efficacy and efficiency. We therefore suggest that manufacturers should consider updating their recommendations. Our analyses further revealed a need for further research on the influence of payload on UAV sprayer swath widths, downwash‐crop interaction and the transposability of swath width measurements to plant protection treatments in vegetated areas. Finally, our data show that lateral wind generated by the UAVs considered is not substantial at distances of 10 m and 20 m and heights of 1 m and 2 m and also not associated with UAV weight. Whether or not this means that UAV weight does not tangibly influence the risk of off‐target drift needs to be investigated in subsequent research. We also observed a strong correlation between the total propeller working area and maximum take‐off weight. Future research should consider the lift coefficient to assess whether the lift required for take‐off and flying of different UAV models from different manufacturers is indeed mainly provided by propellers size rather than propeller speed. To gain insight into agricultural practice under the current regulatory framework, we conducted a survey. The results revealed that plant protection treatments with UAVs have increased substantially between 2018 and 2023. In 2023, up to 11.5 % of Swiss vineyards may have been treated with UAV sprayers and other uses, such as spreading of slug pellets, seem to gain importance as well. Overall, these results suggest that Switzerland's rugged landscape and the presence of small sized agricultural plots may have shaped certain use patterns while others have no obvious link to local topology and/or land use. The advent of UAV sprayers has been accompanied by a decline of the surfaces treated with manned helicopters. Finally, we reviewed the results of efficacy trials performed in Swiss vineyards. These trials suggest that in the context of treatments against fungal pathogens, a main limitation of UAV sprayers is the relatively low amount of spray deposit around and on the bunches. These findings are in agreement with the results of independent trials performed outside Switzerland. In cases of high disease pressure, UAV treatments, especially those involving contact fungicides, should therefore be complemented with additional ground‐based plant protection treatments.

As an outlook, we would like to mention (possible) trends:

- UAV sprayers are subject to on‐going technical changes and improvements. For example, recently released UAV models, such as the XAG P100 Pro and the DJI Agras T50, are equipped with rotary atomizers instead of hydraulic nozzles. In our experience, these trends have improved coverage, a main limitation of the UAV models available in 2018–2020 and used in the efficacy trials described in Results of efficacy trials section. We encourage future research on improving coverage.
- According to the results of our survey, the use patterns associated with UAV sprayers/spreaders have changed over the past years and are likely to continue to do so in the future. For example, weed control could soon commonly involve camera‐ and AI‐based localization of weeds on a plot, followed by herbicide spot applications instead of broadcast applications.
- As UAV sprayers are increasingly used worldwide, the agrochemical industry may develop formulations specifically designed for UAV sprayers. Considering the limited payload of these systems, these formulations would likely require only little water for dilution prior to use if not no water at all.^2^
- Efforts are being made to harmonize the assessment of the risks of UAV sprayers internationally. One example is the on‐going work of the Drone/UASS subgroup of the Organisation for Economic Co‐operation and Development.^28^ The European Precision Application Task Force is also concerned with UAV sprayers.^51^

From a regulatory perspective, it will be crucial to monitor these trends, for example, through regular exchanges with relevant stakeholders, and to respond to them through the generation of new data and the adaptation of the regulatory framework, if appropriate.

## CONFLICT OF INTEREST

None declared.

## Supporting information


**Data S1.** Supporting Information.

## Data Availability

Anonymized data from the technical assessments (Section 3) and the data generated in the efficacy trials (Section 5) will be made available upon request. The raw data of the survey (Section 4) will not be shared.
