# Supplementary material for: Plant protection treatments in Switzerland using unmanned aerial vehicles: regulatory framework and lessons learned
Source: Pest Manag Sci. 2025 Feb 27;81(7):3419–29. doi: 10.1002/ps.8721 (PMC12159360; doi:10.1002/ps.8721)
Supplement: Supplementary file 1 — Data S1. Supporting Information. [file PS-81-3419-s001.docx]

# Supporting Information concerning the methodology of the survey

All individuals holding an authorization for UAV-based plant protection treatments were contacted at the beginning of the season of 2024 regarding their willingness to complete a questionnaire. Authorization holders who had agreed to participate were then asked to fill in a questionnaire to provide information on each UAV-mediated treatment performed so far. This information largely corresponded to the elements that need to be documented in a logbook (see Section 2.3 of the main text: “Authorization holders must document all flights”) and concerned the municipalities in which the plots treated by UAV were localized, their geographical coordinates (if available), their surface areas, the cultivated crop(s), the year(s) during which the treatment(s) was/were performed as well as the type(s) of active substance(s) used.

We intended to aggregate data as follows: (i) The area treated using UAVs per year since 2017, (ii) the area treated using UAVs during the 2023 season by crop, (iii) by target pest, (iv) by canton of the Swiss Confederation, and (v) by slope. Slopes were to be extracted based on the geographical coordinates using the map of agricultural zones and regions of the Federal Office for Agriculture.^1^ However, some respondents did not use the form we provided while others provided data that could not directly be aggregated in the ways described above. In the following, we describe the deviations from the original survey plan that we encountered and how we dealt with them to prevent survey attrition:

- Instead of data relating to individual agricultural plots, six respondents provided data that were (in part) already aggregated. In general, we accepted aggregated data and followed up with the respondents when necessary to ensure that their data could be analyzed as described in the previous paragraph.
- Treatments that were outside the scope of the present work were excluded. In one case, treatments to be excluded were part of an aggregated dataset. It was not clear which crop(s), canton(s) and slope(s) were concerned by the particular treatments to be excluded. It was therefore assumed that all crops, cantons and slopes indicated in the aggregated dataset were equally concerned and the treated surfaces were downscaled accordingly.
- Two respondents did not provide information on the precise location of the plots that were treated but the names of regions or areas spanning multiple cantons. Here, the treated plots were assumed to be located in all cantons concerned and the surfaces were split among them in a weighted manner. Relative weights were derived from the respective cantonal statistics on land use.^2^ For example, if the treatment concerned field crops the total area of land used for growing field crops in the respective canton was used as a weighting factor.
- One respondent indicated that treatments were performed in “a total overflown viticultural area of 220 hectares”. We assumed that the data provided by the respondent were likely to relate to multiple treatments of the same plots. As common treatment schedules of grapevine involve 7–10 fungicide treatments per year in Switzerland, we assumed that a total area of 27.75 hectares were treated that year.
- In many instances, no information on slopes or geographical coordinates that would allow to infer such information were provided. Under these circumstances, we assumed that the data on the slopes of vineyards that were available were representative for those vineyards for which no such information was available and upscaled the former accordingly. For all other scenarios, such as treatments of field crops, we assumed that slopes were <18 %.
- One respondent aggregated slopes as follows: 0 %, <30 %, <50 %, >50 % and additionally indicated the treated surfaces corresponding to terraces. He/she further declared that in cases one part of a plot fell into one of the categories and another part into another category the entire plot was assigned to both categories, and hence, the total indicated surface by slope exceeded that by crop in some cases. We downscaled the surfaces by slope to match the surface by crop. We further assumed that terraces had slopes >30 %. Finally, all data were transformed to match the slope categories of the other datasets. If the data could not be unambiguously assigned to a single category, for example, slopes >30 %, they were assumed to be evenly distributed over all possible categories. In the example given, ⅓ of the treated surface was assumed to display a slope 18–35 %, ⅓ a slope >35–50 % and ⅓ a slope >50 %.

Finally, we decided not to represent the data relating to the season of 2024 in the paper, as that season was still on-going at the time the survey was conducted.

# Supporting Information concerning the efficacy trials

Each trial site covered approximately 1,000 m^2^. To reflect Swiss agricultural practice, five UAV sprayers from three different manufacturers were deployed, namely AgroFly (SpUAV), DJI (Agras MG-1P, MG-1S and T16) and Aero41 (AGv2). Field trials involved three spray schedules, which corresponded to integrated pest management, organic pest management and organic pest management including potassium phosphonate treatments.

The application parameters are indicated in the following table:

| **UAV sprayer** | AgroFly (SpUAV) | DJI Agras  MG-1S | DJI Agras  MG-1P | DJI Agras T16 | Aero41 (AGv2) |
| --- | --- | --- | --- | --- | --- |
| **Nominal volume rate [l/ha]** | 100 | 100 | 100 | 100 | 100 |
| **Nozzle type** | Teejet TXA8004 | Teejet XR110015 | Teejet XR110015 | Teejet XR110015 | Teejet TXA8004 |
| **Nominal flight speed [m/s]** | 2.8 | 2.8 | 2.0 | 2.0 | 2.8 |
| **Nominal flight height above ground [m]** | 4.0 | 4.0 | 3.5 | 3.5 | 3.5 |
| **Nominal swath width [m]** | 2.2 | 3.0 | 3.0 | 3.5 | 3.2 |

It was deemed that no reliable data on control efficacy could be generated from three field trials: In two cases, the disease pressure was very low on the corresponding plots that year and in another case, spray drift towards the control sub-plot occurred at the time a neighboring sub-plot was treated. In the remaining 9 field trials, the frequency and severity of downy and powdery mildew infestations before harvest were assessed on leaves and bunches. Here, the frequency was quantified through visual inspection of 3 × 100 leaves and 3 × 50 bunches, whereby the number of infected organs was determined and expressed as a percentage. In addition, the severity of infection was quantified by estimating the percentage of the surface area affected by the disease and scoring the results as follows: 0 = no symptoms, 1 = >0–2.5 %, 2 = >2.5–10 %, 3 = >10–25 %, 4 = >25–50 %, 5 ≥ 50 %.

Further details on the methodology can be found in ref. 3.

# References

1. FOAG, Karte der landwirtschaftlichen Zonen und Gebiete. Available online: <https://s.geo.admin.ch/6ee4f215a7>, accessed multiple times between June 2 and 21, 2024.

2. FOAG, Agrarbericht 2023. Available online: <https://www.agrarbericht.ch/de>, accessed on July 17, 2024.

3. Dubuis P-H and Jaquerot A, Evaluation of the performance of drone treatments to control downy and powdery mildew in grapevines. *BIO Web Conf*. **50**:01006 (2022). <https://doi.org/10.1051/bioconf/20225001006>.
